# Nanoparticles Loaded Thermoresponsive In Situ Gel for Ocular Antibiotic Delivery against Bacterial Keratitis

**DOI:** 10.3390/polym14061135

**Published:** 2022-03-11

**Authors:** Muhammad Naseer Abbas, Saeed Ahmad Khan, Sajid Khan Sadozai, Islam A. Khalil, Asem Anter, Marwa El Fouly, Ahmed H. Osman, Mohsin Kazi

**Affiliations:** 1 Department of Pharmacy, Kohat University of Science and Technology, Kohat 26000, Pakistan; dr_naseer86@yahoo.com (M.N.A.); cajidkhan@hotmail.com (S.K.S.); 2 Division of Molecular Pharmaceutics and Drug Delivery, College of Pharmacy, The University of Texas at Austin, Austin, TX 78712, USA; 3 Department of Pharmaceutics, College of Pharmacy and Drug Manufacturing, Misr University of Science and Technology, Giza 12566, Egypt; islam.khalil@must.edu.eg; 4 Microbiology Unit, Drug Factory, College of Pharmacy and Drug Manufacturing, Misr University of Science and Technology, Giza 12566, Egypt; asem_micro1980@hotmail.com; 5 Department of Ophthalmology, Research Institute of Ophthalmology, Giza 12211, Egypt; marwa.rio2014@gmail.com; 6 Department of Pathology, Faculty of Veterinary Medicine, Cairo University, Giza 12211, Egypt; ahosman2007@hotmail.com; 7 Department of Pharmaceutics, College of Pharmacy, King Saud University, P.O. Box 2457, Riyadh 11451, Saudi Arabia; mkazi@ksu.edu.sa

**Keywords:** ocular administration, ocular infections, sol–gel transition, poloxamer N-407, gelatin

## Abstract

Antibiotics delivered through conventional dosage against ophthalmic infections show lower therapeutic efficacy due to their low residence time. Therefore, there is a great need to design and develop novel dosage forms that would increase the ocular residence time of antibiotics at the site of infection. This study describes the development of nanoparticles laden in situ gelling solution, intended to sustain antibiotic release for improved therapeutic efficiency. Oxytetracycline-loaded gelatin-polyacrylic acid nanoparticles were prepared and incorporated in poloxamer-N407 solution. The rheological properties of the system were studied concerning time and temperature. Moreover, in vivo biocompatibility of the system was ascertained using the Draize test and histological studies. Finally, the optimized formulation was evaluated for in vitro antibacterial activity against one of the most common keratitis causing bacteria, *Pseudomonas aeruginosa*. Additionally, the in vivo efficacy was evaluated on the rabbit’s eye conjunctivitis model. The formulation showed a sustained effect against keratitis; furthermore, the antibacterial activity was comparable with the commercial product.

## 1. Introduction

An ocular drug delivery system is considered as challenging and difficult as the human eye is an isolated organ where the delivery of drugs is quite difficult. Antibiotics in the form of eye drops are one of the most widely used topical administrations against various eye infections. However, the conventional ophthalmic formulations must be administered very frequently to maintain optimum concentration at the site of infection since the rapid turnover of tears and extensive elimination of drugs through nasolacrimal drainage leads to poor bioavailability of ophthalmic drops [1]. In conditions such as topical ocular infections, the insufficient antibiotic concentration at the site of infection may lead to bacterial resistance and treatment failure [2]. Therefore, it is imperative to maintain optimum antibiotic concentration at the ocular surface without frequent administration to improve therapeutic efficiency and patient compliance.

Infections of the anterior segments of the eye are more common such as conjunctivitis, scleritis, blepharitis, keratitis, etc. The most common ocular infections are mainly caused by bacteria and sometimes by viruses [3]. Bacterial keratitis is the primary ocular infection in children and the second most common ocular infection in adults, followed by bacterial keratitis [4]. If these infections are not treated properly, these may cause visual impairment and ultimately visual loss [5]. Staphylococcal species and *Pseudomonas aeruginosa* are some of the most common agents in causing bacterial conjunctivitis and keratitis [6]. Various studies have shown the presence of *Pseudomonas aeruginosa* in bacterial keratitis, with more progressiveness and large infiltrate and scarring [7]. Antibiotics such as Cephalosporin, Aminoglycosides, and Tetracyclines have been used to treat ocular infections [8]. Tetracyclines (oxytetracycline) are largely used for a variety of ocular infections. In ophthalmology, oxytetracycline and other tetracycline derivatives have been extensively investigated for anti-protease activity, antimicrobial activity, and anti-angiogenic properties as well as their effects on corneal epithelial cell proliferation [9]. These antibiotics have been administered topically in the form of eye drops, ointments, and gels for the treatment of various ocular infections. However, due to the unique anatomy and physiology of the eye, various problems including low efficacy and low drug bioavailability have been associated with these topically administered drugs [10].

The treatment efficiency of topically applied antibiotics against ophthalmic infections can be improved by increasing their ocular residence time. Several approaches have been adopted for increasing the corneal residence time of drugs such as microparticles, gels [11], gel-forming solutions [12], micelles [13], liposomes [14], nanoemulsions and nanoparticles [15], etc. One of the most promising approaches in increasing the residence time at the ocular surface is to reduce nasolacrimal drainage. This is typically achieved by increasing the viscosity of the vehicle [16]. Moreover, ocular inserts can also provide the enhanced residence of the drug at ocular surface. However, foreign body sensation associated with ocular inserts leads to low patient compliance. Alternatively, in situ gel forming solutions have been proven to be one of the most promising systems in increasing the ocular residence time of drugs without posing any deleterious effects [17]. In situ gel forming solutions are applied in the form of solutions, which undergo sol-to-gel transition after administration into the eye. The gel formation can be triggered by various mechanisms such as changes in temperature (thermosensitive gel), alteration in pH (pH-sensitive gel), and ionic strength (ionic gelation) [18]. Thermoresponsive gelling systems are polymeric solutions that change from a solution to gel when exposed to temperature changes. Several natural and synthetic polymers demonstrate thermoresponsive gelling properties at body temperature [19]. For instance, chitosan-based thermoresponsive hydrogels were introduced by Chenite et al. for the sustained delivery of latanoprost and ferulic acid to rabbit eyes with good biocompatibility properties [16]. Furthermore, poly *N*-isopropyl acrylamide (pNIPAAm) is also one of the well-established thermoresponsive polymers with a gelation temperature of ~32 °C, which can be tuned using hydrophilic monomers [18]. Poloxamers are synthetic amphiphilic polymers exhibiting good thermoresponsive behavior. Moreover, poloxamer solutions are biocompatible and can form transparent gels that do not obstruct normal vision. Hence, they are widely used for ophthalmic drug delivery systems [20].

In recent years, the development and evaluation of a new drug delivery system consisting of drug-loaded NP laden in situ gel have shown enhanced therapeutic efficacy and ocular bioavailability [21]. Enhanced ocular bioavailability of hydrocortisone-loaded poly(lactic-co-glycolic acid (PLGA) NPs incorporated in thermoresponsive in situ gel was observed [22]. Improved precorneal residence time and enhanced ocular bioavailability was observed for Sparfloxacin-loaded PLGA-NPs incorporated in pH-sensitive in situ gel [23]. PLGA-NPs of a pro-drug of ganciclovir loaded in thermoresponsive PLGA-PEG in situ gel showed enhanced efficacy for corneal keratitis [24]. Bioavailability of water-soluble drugs can be enhanced by formulating NP laden in situ gel [25]. Thermoresponsive in situ gel is the most widely used ocular drug delivery system due to the easy availability of many safe and biocompatible thermoresponsive polymers and their easy gelation at body temperature. Among them, poloxamer is one of the most widely used polymers. Poloxamer exhibits a very finely tunable T_sol-gel_, thus making it a suitable candidate for use in pharmaceutical and biomedical applications [26]. Poloxamrer 407 has been approved as an “inactive ingredient” by FDA for a wide range of products including suspensions, solutions, intravenous, ophthalmic, and inhalation formulations [27]. During an in vivo rabbit eye model study, significantly increased drug absorption of poloxamer-based in situ gels were observed for loteprednol, methazolamide, and timolol compared to the standard formulations [28].

From the last few decades, different natural and synthetically prepared polymers have been extensively used for NP based ocular drug delivery [29]. Among these polymers, polyacrylic acid (PAA) has been used extensively due to its muco-adhesive properties [25] and safety to surrounding tissues due to its non-abrasiveness and flexible nature [30]. Recently, biopolymer-based NPs have gained in popularity and interest due to their unique enhanced bioavailability, biodegradability, and sustained release effects in ophthalmic drug delivery [31]. Gelatin is one of the most widely used natural biopolymers due to its easy and low-cost availability, the presence of large active groups, mucoadhesion, biocompatibility, and biodegradable nature [32,33,34,35,36]. However, the problem with gelatin-based NPs is their need to use cross linkers [31,32], which usually have the potential for reactivity and toxicity [37]. In our previous study, we exploited the phenomenon of poly-electrolyte complexation (PEC) [38] for the formation of gelatin and PAA NPs without the use of toxic cross linkers [39].

In this context, we developed poloxamer-based thermoresponsive in situ gel forming solutions for the delivery of oxytetracyclin loaded PAA-gelatin NPs with the objective to sustain the release of antibiotics for improved therapeutic efficiency.

## 2. Materials and Methods

### 2.1. Materials

Poloxamer-N407 was taken from BASF (Ludwigshafen, Germany). Poly acrylic acid (PAA) and gelatin type B were purchased from Sigma-Aldrich (St. Louis, MO, USA). Oxytetracycline HCl was purchased from Shenzhen Nexconn Pharmatechs Ltd. (Shenzhen, China). Polyvinyl alcohol (PVA) was obtained from Merck KGaA (Darmstadt, Germany). All other solvents and reagents used were of analytical grade.

### 2.2. Methods

#### 2.2.1. Preparation of Nanoparticles

Oxytetracycline-loaded gelatin-PAA NPs were prepared according to our previously reported method [39] with slight modification. In brief, 5 mg of oxytetracycline and 10 mg gelatin were dissolved in 1 mL water at 50 °C, and the solution was added drop wise to a 4 mL dispersion medium composed of a water—ethanol mixture (1:2) containing 15 mg of PAA and specific amounts of the stabilizer (PVA 2% *w*/*v*). Spontaneous formation of NPs took place through the polyelectrolyte complexation (PEC) phenomenon. For the optimization of NPs, various parameters including stabilizer concentration, gelatin concentration, dispersion medium composition, and drug/polymer ratio were investigated according to our previously reported work [39].

#### 2.2.2. Physico-Chemical Characterization of Nanoparticles

Prepared NPs were characterized for particle size, PDI, surface morphology, zeta potential, and drug–excipient interaction by following our previously reported method [39]. To determine the mean particle size and PDI of NPs, the dynamic light scattering (DLS) method was used. Zeta potential determination was carried out through the dip cell cuvette method [40]. Determination of the surface morphology of NPs was conducted through scanning electron microscopy (SEM).

#### 2.2.3. Optimization of Vehicle for In Situ Gel System

The cold method was used for the preparation of poloxamer-N407 solutions [41]. Required quantities were taken and dissolved in distilled water under continuous agitation on a magnetic stirrer in a glass beaker, maintaining a temperature at 5 °C ± 3 °C. Stirring was continued for around two hours until the formation of a homogenous solution [42]. This solution was kept in refrigerator for 24 h at 5 °C ± 3 °C in order to obtain a clear solution. The concentration of poloxamer-N407 was varied and the rheological properties with respect to temperature were investigated.

#### 2.2.4. Preparation of Nanoparticles Laden In Situ Gel System

Drug-loaded NPs were dispersed in a poloxamer solution. Preservative (0.01% *w*/*v* benzalkonium chloride) was added to this mixture. The pH of the formulation was maintained at 7.4. The mixture was mixed continuously for 30–40 min.

### 2.3. Characterizations of Nanoparticle Laden In Situ Gel System

#### 2.3.1. Gelation Temperature

Gelation temperature determination in simulated tear fluid (STF) was carried out according to the method by Cao et al. [43]. Briefly; the gel forming solution was stirred continuously at 50 rpm and temperature was gradually increased from 25 °C to 50 °C using a water bath. Gelation temperature was noted at the point where the stir bar stopped stirring.

#### 2.3.2. Rheological Studies

Rheological properties of the prepared solutions and gels were studied using a Brookfield synchroelectric viscometer (RVT model). The prepared solution was placed in a small adaptor of the viscometer. To determine the viscosity at different speeds, angular velocity was gradually increased from 10 to 100 rpm. The hierarchy of angular viscosity was also reversed. Viscosity was measured by taking the average of two readings [44].

#### 2.3.3. pH Determination

pH plays a very important role in ophthalmic formulations as the eye can tolerate formulations within a specific pH range (6.5–8.5) [45,46]. Therefore, pH determination is a very crucial and important parameter in ophthalmic preparations. A calibrated pH meter (Mettler Toledo, Zürich, Switzerland) was used to determine the pH of the formulations. Triplicate values were taken and pH was calculated as the average of these values.

### 2.4. In Vitro Drug Release

In vitro drug release studies of the in situ gel solutions were performed using the dialysis bag method, as reported previously [39]. This study was conducted in a simulated tear fluid consisting of sodium bicarbonate 0.22 g, sodium chloride 0.68 g, calcium chloride dehydrate 0.008 g, and potassium chloride 0.14 g, dissolved in 100 mL distilled de-ionized water [47,48]. The study was conducted at 50 rpm, maintaining a temperature of 34 ± 0.5 °C, and all readings were taken in triplicate [49].

### 2.5. In Vivo Biocompatibility Study

#### Draize Test

The Draize modified test was carried out to determine ocular irritation in the eyes of white albino rabbits after application of the formulation [50]. A dose of 0.01 mL in situ gel (containing 0.2% oxytetracycline) was administered into the lower cul-de-sac of the left eye of each rabbit every 30 min for six hours, with the right eye serving as the control. Eyelids were gently kept together for around 10 s to prevent the loss of the instilled preparation. After dose instilment, the rabbit’s eyes were observed for any possible ocular reactions including redness of the eyes, conjunctival chemosis, discharge, and corneal and iris lesions. These observations were conducted at regular time intervals of 1, 24, 48, 72 h, 7, 14, and 21 days. The assessment was conducted according to the histological grading system for eye irritation evaluation. Different grading from 0 to 3 were carried out on the basis of the absence or presence and severity of symptoms including inflammation, redness, and tearing, as shown in Table 1 [51].

The total clinical evaluation scores were added together over the observation time periods to obtain the overall ocular irritation index (Iirr). In any category, a score of 2 or 3 or Iirr > 4 was determined as clinically significant irritation [25].

### 2.6. Histological Evaluation

After 21 days of experiments, tissue specimens were collected from the rabbits’ eyes for histological evaluation. The globes were removed from all rabbits and these were fixed in Davidson’s solution. The eye was kept submerged in this solution after enucleating and trimming. After one to two days, the eye was removed from the solution and placed in 10% formalin. Tissue specimens were dehydrated in ascending grades of alcohol. Next, these were cleared in xylene, embedded in paraffin blocks, and sectioned at a thickness of 4–6 µm. The tissue sections were then deparaffinized with xylol and stained for histological analysis with hematoxylin and eosin (H&E) [51].

### 2.7. Therapeutic Potential of In Situ Gel System

#### In Vivo Antimicrobial Activity

In this study, twelve New Zealand albino rabbits weighing from 2.8–3.5 kg were randomized into three groups (F1, F2, and F3) (Table 2).

All experiments were carried out according to the Guide for the Care and Use of Laboratory Animals. All animals were housed in light controlled standard cages at 20 ± 1 °C without any restriction to food and water. Approval of protocols was conducted by the Ethical Committee of Misr University of Science and Technology (Giza, Egypt, PT10-2020; Date of approval: 23 August 2020) and the experiments were carried out under the supervision of qualified veterinarians. Each eye was individually infected by log phase culture of the clinical isolate of *Pseudomonas aeruginosa* (2 × 10^8^ CFU/mL). After local anesthesia with proparacaine HCl (Alcaine^®^, Alcon), each rabbit’s cornea was injected with 0.1 mL bacterial suspension using a 30-gauge needle. Bacterial keratitis was confirmed after 12 h of inoculation, and topical therapy was initiated. Each formulation was applied twice daily into the left eye of each rabbit, while the right eye served as the control (non-treated).

On a daily basis, the eye balls were evaluated for various symptoms such as conjunctival congestion, chemosis, mucopurulent discharge, and palpebral fissure before and after drug administration. At the end of the experiment, histopathological examination of hematoxylin and eosin-stained tissues (H&E) were conducted according to the previously mentioned protocol in Section 2.6 Furthermore, the CFU of bacteria load were calculated in eye tissue after infection with *Pseudomonas aeruginosa* with and without treatment by antibacterial formulations. The infected and treated eyes of each group of rabbit were divided into the anterior and posterior section using sterile scissors. Dissected tissues from the eyes were homogenized in 5 mL of 0.9% saline. For CFU determination, 10 μL of each homogenized tissue was subjected to 1:10,000 dilution in sterile saline. Afterward, 100 μL of each diluted sample was placed on a Nutrient agar plate and incubated at 37 °C for 24 h. Plates with bacterial colonies ranging from 30 to 300 were counted and the average results of each cornea were reported as CFU/mL. Bacteria count in the sample used to induce keratitis was 2 × 10^8^ CFU/mL [52].

$$\mathrm{CFU}/\mathrm{mL}=\frac{\mathrm{number}\;\mathrm{of}\;\mathrm{colonies}*\mathrm{dilution}\;\mathrm{factor}}{\mathrm{Volume}\;\mathrm{of}\;\mathrm{culture}\;\mathrm{plate}}$$

### 2.8. Statistical Analysis

For statistical analysis, all data obtained were expressed as mean ± standard deviation. To compare the results, one-way analysis of variance (ANOVA) was used. Data were considered significant at a *p*-value < 0.05 using Graph Pad Prism software (Version 5).

## 3. Results and Discussion

### 3.1. Physico-Chemical Properties of Nanoparticles

Generally, the particle size for ophthalmic preparations must be smaller than 10 microns [53]. Due to the large surface area and sustained release properties, NPs can potentially be used for optimum therapeutic efficacy [54].

**Figure 1:**
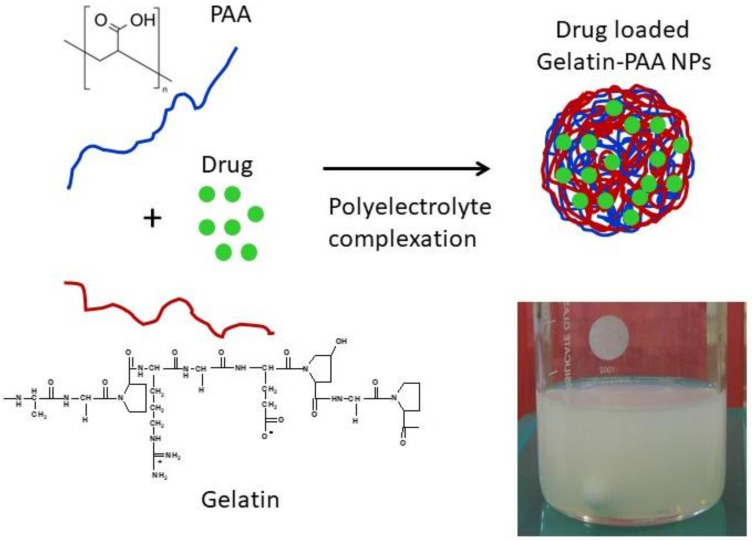
Schematic representation of oxytetracycline-loaded gelatin-PAA nanoparticles prepared by polyelectrolyte complexation between gelatin and PAA (adopted from our previous publication [39]).

Oxytetracycline-loaded gelatin-PAA nanoparticles were prepared by the polyelectrolyte complexation phenomenon according to our previously reported method (Figure 1). Particle size is a very important parameter in NP formulations as it may greatly influence the dissolution and bioavailability of drugs [55]. Various parameters have been evaluated to obtain stable, small, and mono-dispersed NPs [39]. PVA was used as a stabilizer for the stability of NPs. Different parameters such as PVA concentration, dispersion medium composition, and gelatin concentration on NP size and polydispersity were critically evaluated. These results were reported in our previous research paper [39]. For instance, increased gelatin concentration showed an increase in the size of NPs and vice versa. At the same time, an increase in gelatin concentration showed very little effect on PDI values. An increase in NP size with an increase in gelatin concentration is in line with the previously published data [39]. Zeta potential values also play an important role in the stability and in vivo therapeutic effects of NPs. For the stability of NPs, zeta potential values must be above +25 mV or less than −25 mV [56]. Our study revealed an increase in zeta potential value with increased ethanol concentration in the dispersion medium. The highest zeta potential values ranging from −72.3 to −68.4 mV was observed for blank NPs. Zeta potential values ranging from −40.2 to −63.4 mV was observed for drug-loaded NPs, while a decrease in zeta potential values of NPs was observed with an increase in drug loading [39].

**Figure 2:**
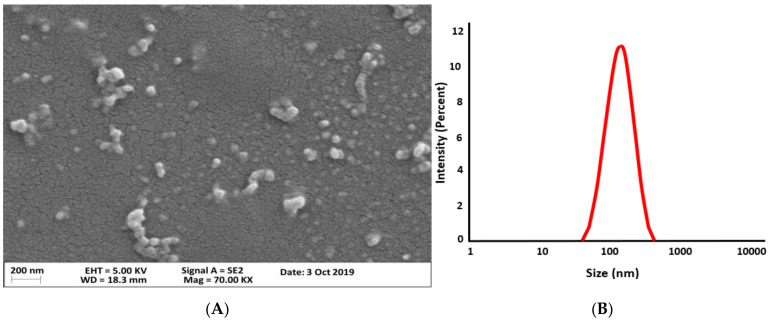
SEM micrograph (**A**) and dynamic light scattering (DLS) data (**B**) of gelatin-PAA NPs, (particle size distribution data is reproduced from our previous article [39]).

The spherical shape of drug-loaded NPs was revealed by thee SEM micrograph (Figure 2A), while the DLS size was much larger than that in SEM (Figure 2B), which may be due to the fact that DLS measures the hydrodynamic diameter of NPs [57].

### 3.2. Physico-Chemical Characterization of In Situ Gel

Poloxamer-N407 thermoresponsive in situ gel was prepared successfully using the cold method. The cold method is one of the most widely used and preferred methods as it provides a clear solution for in situ gel [58]. Several solutions with different poloxamer concentrations were prepared (i.e., 16%, 17%, 18%, 19% and 20%). The solutions were refrigerated for 24 h to obtain a clear solution.

**Figure 3:**
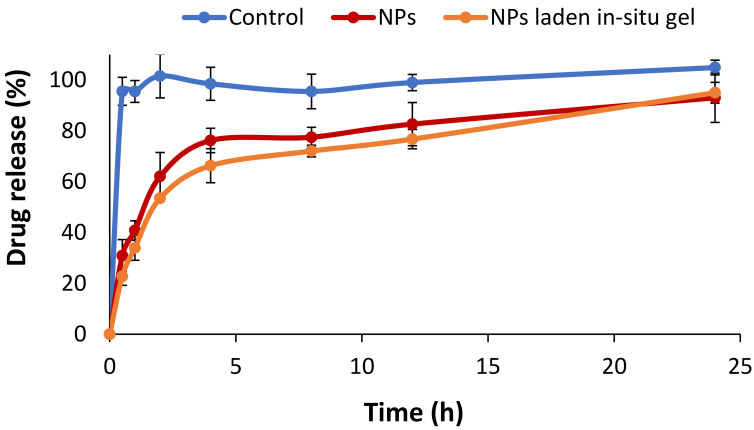
In vitro drug release profile of drug loaded NPs, NP laden in situ gel and pure drug from the dialysis membrane in STF (at 37 ± 2 °C).

Physicochemical characterization of in situ gel formulation is of prime importance and must be considered while formulating in situ gel. In particular, it is of prime importance when these formulations are intended for ocular administration. Clarity is one of the crucial and important factors that must be evaluated for ophthalmic preparations [59]. Clarity of all developed formulations was evaluated by visual observation against a black and white background. The results showed that all formulations had sufficient and proper clarity. In vitro drug release profiles of the drug loaded NPs, NP laden in situ gel, and pure drug from dialysis membrane in STF were conducted at 37 °C (Figure 3). In the case of the pure drug, an abrupt maximum drug release within half an hour was observed, showing no controlled drug release. While in the case of the drug loaded NPs and NP loaded in situ gel, a biphasic drug release profile was observed as a significant amount of drug was released from these formulations in the first two hours, followed by a gradual increase in the drug release pattern in the later hours. For instance, in the case of drug loaded NPs, 30% drug release was observed in the first half hour of the experiment followed by 62% drug release in two hours. Maximum drug release of 93% was observed after 24 h. The NP laden in situ gel showed comparatively good drug release behavior that was comparable with drug loaded NPs. In the first half hour, 22.7% of drug release was observed, followed by 53.4% of drug release in two hours, while the maximum drug release of 94% was observed after 24 h. This controlled drug release from NPs and NP laden in situ gel can be attributed to a tighter gelatin matrix in NPs and sufficient crosslinks in the polymer matrix that could hinder the release of the drug from the polymer, thus enabling a controlled drug release pattern [60].

Gelation temperatures of in situ gel formulations with and without drugs were also evaluated to be one of the important physicochemical characteristics. Gelation temperatures for all formulations were recorded in the range of 28.3 ± 0.15 °C to 31.2 ± 0.32 °C. This shows that all of the formulations can readily be converted to gel, once they are instilled into the eyes (Table 3).

The pH of the formulation was also evaluated as it is significant for patient compliance. Formulations with pH values beyond 4–8, may cause discomfort and irritation to the eye. Furthermore, bioavailability of the drug can be further decreased due to irritation and increased tearing [61]. The pH of the prepared formulations with and without drugs were evaluated with the help of a calibrated pH meter. pH values for all preparations were found in the range of 7 to 7.45, which revealed the non-irritant nature of the formulations. Tear fluids in the eyes have the potential of diluting and buffering capability of added substances in small volumes, hence, the eye can tolerate a wide pH range. The pH range for ophthalmic preparations is from 6.5 to 8.5 [45,46].

### 3.3. Effect of Poloxamer Concentrations on Rheological Properties

Ophthalmic formulations must have viscosity in the range of 25–50 cp [62,63] in order to be easily instilled into the eye, which would later on undergo sol–gel transition due to eye temperature exposure. After instillation of these in situ solutions, these were transformed into gel within a few seconds of instillation (Table 3). Additionally, the gel formed must preserve its integrity without dissolving or eroding for longer periods of time to provide sustained release of the drug within the eye [64]. The effects of different poloxamer concentrations on the viscosity of the gel were evaluated at different temperatures. The viscosity of the formulation was found to increase with the increasing poloxamer concentration. The findings of Patil et al. are in line with these findings [61]. The results are shown in Table 4, indicating that gel viscosity is in direct relation to the poloxamer concentration. Higher poloxamer concentration (i.e., 20% and 19%) resulted in a solution that had a very high viscosity value at room temperature. The viscosity increased substantially after gelation. On the other hand, poloxamer concentrations of 18%, 17%, and 16% exhibited a viscosity of 83.7 cP, 29.4% cP, and 17.3 cP, at room temperature, respectively. These results matched those reported by Chaudhry et al. [62].

**Figure 4:**
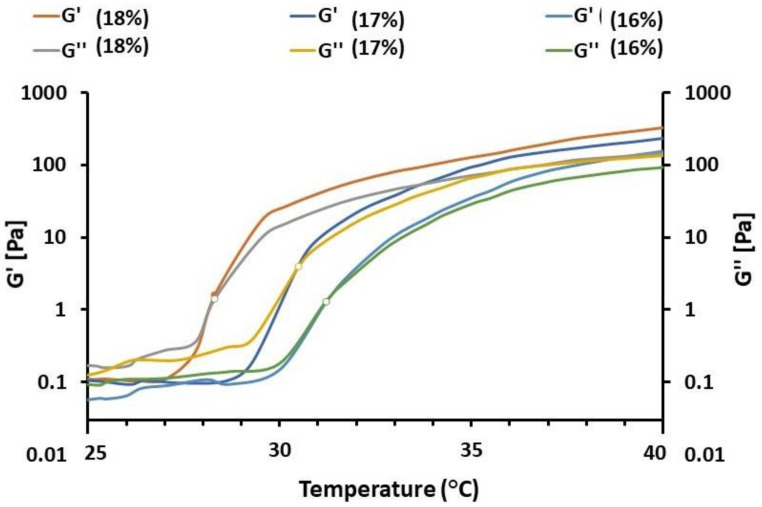
The impact of poloxamer concentration on gel formation.

Three lots of gel with 18%, 17%, and 16% poloxamer concentrations were used for rheological evaluations. Temperature sweeps were performed to obtain the T_sol-gel_ for different polymer concentrations. Elastic modulus (G’) and viscous modulus (G”) for different concentrations of poloxamer were evaluated as a function of temperature at a frequency of 0.01 Hz, as shown in Figure 4. The gelation temperature was identified as the temperature at which the G′ and G″ curves intersect each other [62]. The T_sol-gel_ for different lots of gels with different poloxamer concentrations were recorded to be different. Increased poloxamer concentration (18%) in the gel reduced the T_sol-gel_ from 31.2 °C to 28.3 °C, while the low poloxamer concentration (16% *w*/*v*) in the gel showed a relatively high T_sol-gel_ of 31.2 °C. It is also clear that G′ is affected by temperature variations. At low temperature and low poloxamer concentration, G′ is quite low for a solution. However, as temperature and poloxamer concentration increased, the value of G′ increased because of the gel formation. Figure 4 indicates that once sol–gel transition has occurred, then the value of G′ becomes independent of any change in temperature. Edsman et al. reported similar results in their study [44].

### 3.4. In Vivo Evaluation

#### 3.4.1. Ocular Irritation Test

**Figure 5:**
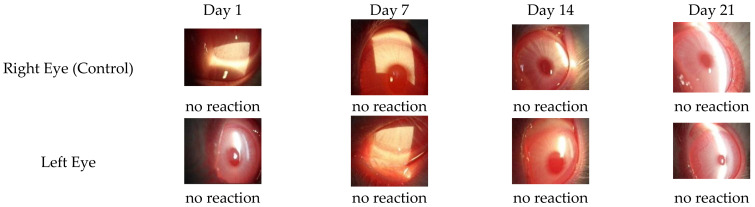
In vivo biocompatibility evaluation of in situ gel in rabbit eyes.

**Figure 6:**
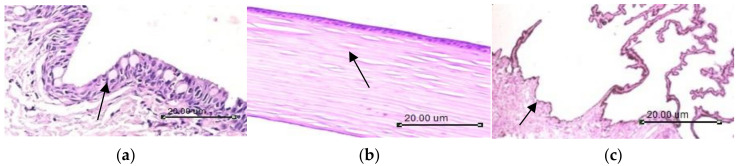
Photomicrograph of (**a**) conjunctiva showing intact epithelial lining arrow; (**b**) cornea showing normal histological structure arrow; (**c**) ciliary body showing normal histological structure arrow (H&EX200).

Previously reported data suggest that in situ gels, in most cases, do not cause irritability or any unwanted effects on the eye [65,66]. The Draize rabbit eye test was performed on New Zealand white rabbits. After administration of the drug loaded in situ gels, no ocular damage or clinical abnormalities were seen in the conjunctiva, cornea, and iris. Only grades 0 and occasionally grade 1 results (mild redness) were observed (Figure 5). No differences were observed between the control (untreated) and treated eyes of each rabbit group. These results showed excellent ocular tolerance of these in situ gels. These findings are consistent with previously published data [38]. All of the examined specimens showed normal histological structure of the conjunctiva, cornea, and ciliary body (Figure 6).

#### 3.4.2. In Vivo Efficacy Study

**Figure 7:**
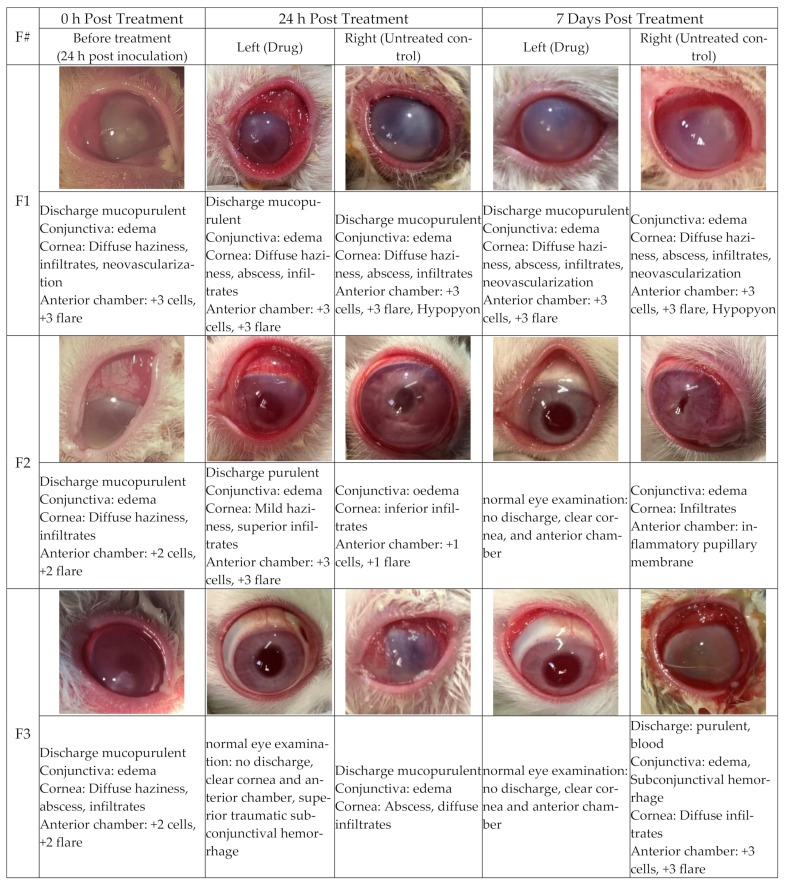
In vivo evaluation of different formulations in rabbit eyes.

For in vivo evaluation of the formulations, New Zealand albino white rabbits were used because of their large sized eyes and well-described anatomy and physiology. Moreover, they are most widely used because of their easy handling, easy and inexpensive availability [66]. These animals were divided into three groups (F1, F2, and F3) with four animals in each group and each group received a particular formulation in specific concentrations, as shown in Table 2. The effects of the drugs were assessed by examining the healing and ocular inflammatory symptoms such as conjunctival congestion, mucopurulent discharge, chemosis, and palpebral fissure on a daily basis before and after the drugs were administered. Left eyes were treated with drugs in all groups, while right eyes served as the untreated control. Bacterial keratitis with symptoms of mucopurulent discharge, conjunctival edema, and cornea with diffuse haziness, abscess, and infiltrates were observed in all groups before administering medicines after inoculation with *Pseudomonas aeruginosa* (2 × 10^8^ CFU/mL). Furthermore, all groups were observed with dead cells and flares in the anterior chambers. The untreated group (F1) showed typical infection symptoms, as shown in Figure 7. Twenty-four hours post-treatment in the case of oxytetracycline-NPs gel (0.2%), most of the symptoms were still present in the F2 group, indicating that oxytetracycline-NPs essentially have no activity against conjunctivitis after 24 h. However, at seven days post treatment, all of these symptoms had vanished, and completely normal eyes with no inflammation, discharge, and a clear cornea were observed. The clear anterior chamber was observed in all rabbits in this group. These results showed complete eradication of conjunctivitis with an oxytetracycline-NP loaded gel (Figure 7). After 24 h of treatment with a commercially available oxytetracycline ointment (0.5%), significant outcomes were observed in the F3 group, indicating that the product was effective (Figure 7).

Furthermore, a full conjunctivitis cure was noted in all eyes after seven days of treatment in this group. These findings demonstrated the product’s ability to treat conjunctivitis (Figure 7). All of these results suggested good in vivo oxytetracycline activity against *Pseudomonas aeruginosa,* which are in good agreement with the previously reported data [67].

According to the above findings, the oxytetracycline NP gel did not exhibit significant results after 24 h of treatment, but did show significant results after seven days, thus showing their sustained release nature. Similar results have been observed by other researchers such as the study of Nagavarma et al. [68]. Moreover, these drug-loaded NPs showed a complete eradication of conjunctivitis even at low drug concentration (0.2%) compared to the 0.5% ointment, indicating that the efficacy of the drug was enhanced in the NP formulation. While in the case of commercially available ointment, significant results were detected even after 24 h, indicating that they did not have sustained releasing effects.

#### 3.4.3. Microbiological Evaluation

**Figure 8:**
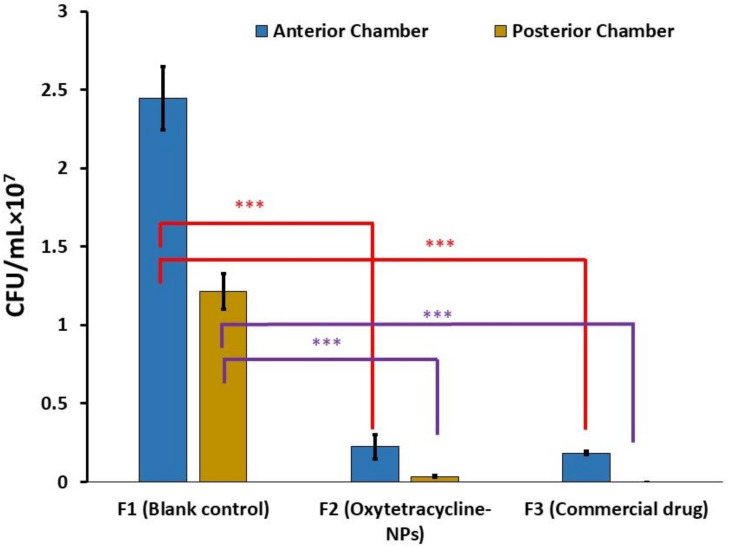
Count of bacteria in eye tissues after infection and treatment in different groups (*** *p* < 0.001).

CFU of bacteria load in ocular tissue following infection with *Pseudomonas aeruginosa* with and without antibacterial formulation treatment was assessed in this study. Under sterile conditions, each group of rabbits’ infected and treated eyes were cut into anterior and posterior sections using scissors. Results are shown in Figure 8. Significant differences were examined by one-way analysis of variance (ANOVA) followed by Tukey’s post hoc tests (* *p* < 0.05, ** *p* < 0.01, *** *p* < 0.001, and **** *p* < 0.0001) using the software Graph Pad Prism software version 6. Untreated eyes served as a negative control. Maximum CFU up to 2.446 × 10^7^ was observed in the anterior chamber of an untreated eye. In the anterior chamber of the eye, F3 (commercial ointment) had the highest antibacterial activity with 0.185 × 10^7^. In contrast, the oxytetracycline-NP gel showed slightly lower activity, indicating that some of the antibacterial properties of the drug might be lost during NP formulation and sustained release of the drug from these NPs. While in the case of the posterior segment of the eye, the best results were seen in the case of F3 followed by F2. These results showed good activity of oxytetracycline against *Pseudomonas aeruginosa*. Khalil et al. and Pervez et al. also reported good efficacy of tetracycline against Pseudomonas species, which supports our results [69,70]. Similarly, in another study, good efficacy of tetracycline against *Pseudomonas* species was reported [71].

### 3.5. Histological Evaluation

Histopathological evaluation of the conjunctival and corneal layers and ciliary body specimens was performed to evaluate and compare the treatment efficiency for oxytetracycline NPs and the commercial drug (Terramycin^®^). Evaluation of the negative control (non-infected cornea), positive control (infected cornea), and corneas treated with drugs were performed. The corneal bacterial infection has been reported to occur in two-steps. Initially, bacteria interact with the surface of the epithelial cells of the cornea followed by the penetration of bacteria into the stroma, resulting in the release of toxins. These toxins are responsible for causing tissue inflammation and damage. Bacterial keratitis is characterized by significant bacterial replication as well as severe ophthalmic changes such as corneal epithelial cell loss, stromal edema, and polymorpho-nuclear leukocyte migration to the tear film [72].

**Figure 9:**
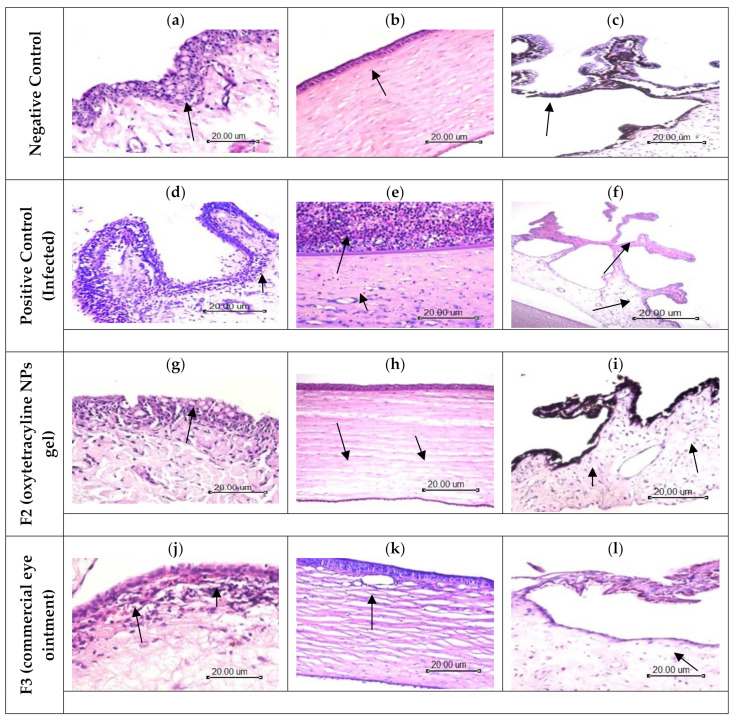
Histological evaluation of the in vivo efficacy study. Photomicrograph of (**a**) conjunctiva showing intact epithelial lining arrow; (**b**) cornea showing normal histological structure arrow; (**c**) ciliary body showing normal histological structure arrow (H&EX200). Photomicrograph of (**d**) conjunctiva showing sloughing of the epithelial lining and massive leukocytic infiltration arrow; (**e**) cornea showing massive leukocytic infiltration, necrosis and vascularization arrow; (**f**) ciliary body showing depletion of pigmented cells and subepithelial edema and leukocytic infiltration arrow (H&EX200). Photomicrograph of (**g**) conjunctiva showed regenerated epithelial cells with goblet cells and few subepithelial leukocytic infiltration arrow; (**h**) cornea showing mild to moderate edema arrow; (**i**) ciliary body showing subepithelial edema and few leukocytic infiltration arrows (H&EX200). Photomicrograph of (**j**) conjunctiva showing slight regeneration of epithelial layer with subepithelial congestion and leukocytic infiltration arrow; (**k**) cornea showing moderate edema arrow; (**l**) ciliary body showing subepithelial edema and few leukocytic infiltration arrows (H&EX200).

Histological sections of the control animals’ (negative control) eyes demonstrated a normal histological structure of the conjunctival layer made up of non-keratinizing stratified squamous epithelium with abundant goblet cells. The conjunctival stroma is a thin, richly vascularized layer enclosing scattered lacrimal glands, plasma cells, macrophages, and mast cells, as shown in Figure 9a. The cornea showed a normal histological structure (Figure 9b). The ciliary body composed of two epithelial cell layers, the non-pigmented and the pigmented epithelium rest on vascular stromal connective tissue (Figure 9c). No lesions were observed in the negative control group (Table 5).

Tissue section of the infected eye with *Pseudomonas aeruginosa* showed suppurative conjunctivitis portrayed by desquamation of the epithelial lining, ulceration, and massive infiltration with neutrophils and macrophages. The subepithelial stroma revealed edema and congestion of blood capillaries (Figure 9d). The cornea showed ulceration of the corneal epithelium with intensive neutrophil cell infiltration, degeneration, and necrosis with subepithelial edema. The stroma showed focal areas of liquefactive necrosis represented by a homogenous structure less basophilic mass. The stroma appeared dispersed as 21nd intensively infiltrated with polymorphous nuclear. Proliferation of keratocytes and new vascularization were noticed in the stroma (Figure 9e). The ciliary body showed a depletion of pigmented cells and leukocytic infiltration and edema of subepithelial stroma (Figure 9f). Severe to moderate lesions were observed in the positive control group (Table 5).

The animal group treated with oxytetracycline-NPs (F2) showed a mild inflammatory reaction compared to the other groups. The conjunctiva showed regeneration of the epithelial lining with goblet cells. Few subepithelial leukocytic infiltration, mainly macrophages and few neutrophils, were seen (Figure 9g). The corneal stroma showed edema and dispersion of connective tissue stroma (Figure 9h). Subepithelial edema and a few leukocytic infiltrations were observed in the ciliary body (Figure 9i). These results suggest the enhanced efficacy of oxytetracycline-NPs compared to other formulations. These results are in consistent with those reported for some other naturally occurring polymers [73,74]. This enhanced activity may be due to overcoming some of the ocular barriers and decreased resistance of the aqueous tear layer by these NPs, hence improving the drug bioavailability and efficacy [75]. Mild to moderate lesions were observed in the NP group (Table 5).

On the other hand, the animal group treated with Terramycin^®^ (F3) showed regeneration of the conjunctival epithelial lining without goblet cells with congestion, edema, and leukocytic infiltration of subepithelial connective tissue stroma (Figure 9j). The corneal layer showed marked edema and the dispersion of stroma (Figure 9k). The ciliary body was observed with subepithelial edema and leukocytic infiltration (Figure 9l). Mild to moderate lesions were observed in this group (Table 5).

The aforementioned results show the different degrees of healing for various formulations used to treat the pathological alterations produced by bacterial infections. The highest and best efficacy was observed for oxytetracycline NPs, followed by the commercially available drug (Terramycin^®^).

## 4. Conclusions

In this study, our previously prepared and reported oxytetracycline loaded gelatin-polyacrylic acid NPs were incorporated in a poloxamer-based thermoresponsive in situ gel. These were evaluated as carriers for sustained ocular drug delivery. The in situ gel of oxytetracycline-loaded NPs was successfully prepared using the cold method. Various physicochemical properties of the in situ gel such as pH, gelation temperature, clarity, and viscosity were evaluated and found to be satisfactory. The rheological properties of the gel and T_sol-gel_ were found to be greatly influenced by poloxamer concentration. Increased poloxamer concentration resulted in a viscous and stable gel and a decrease in T_sol-gel_ resulted in an increased ocular contact time. Developed in situ gel showed good and sustained release efficacy against *Pseudomonas aureginosa*. Excellent ocular tolerance of the formulated in situ gel was observed on the basis of the Draize rabbit eye test. In conclusion, Poloxamer-N407 based thermoresponsive in situ gels could potentially and successfully be employed as sustained-release ocular delivery systems for various drugs.

## Figures and Tables

**Table 1:** Histological grading system for eye irritation evaluation [25].

Scheme	Description
0	No redness, no inflammation, or excessive tearing
1	Mild redness with inflammation and slight tearing
2	Moderate redness with moderate inflammation and excessive tearing
3	Severe redness with severe inflammation and excessive tearing

**Table 2:** Different groups of rabbits receiving the drug formulations.

Formulation	Description	Drug Concentration
F1	Blank control	NPs with no Oxytetracycline
F2	Oxytetracycline-NP gel	Oxytetracycline (0.2% *w*/*v*)
F3	Oxytetracycline eye ointment (Terramycin)	Oxytetracycline 0.5% *w*/*w*

**Table 3:** Gelation temperature and gelation time for different formulations.

Poloxamer Concentration (% *w*/*v*)	Gelation Temperature (°C)		Gelation Time (Seconds)
	Rheometer	Manually Measured	
18%	28.3 ± 0.15	29.5 ± 0.11	6.9 ± 0.01
17%	30.5 ± 0.22	29.3 ± 0.27	9.3 ± 0.05
16%	31.2 ± 0.32	33.1 ± 0.21	30.0 ± 0.07

**Table 4:** The impact of poloxamer concentration on the viscosity.

Poloxamer (% *w*/*v*)	Viscosity (cP)	
	Room Temperature	At Gelation Temperature
20%	18.1 × 10^3^	6.8 × 10^5^
19%	12.3 × 10^3^	5.3 × 10^5^
18%	83.7	6.3 × 10^3^
17%	29.4	2.4 × 10^3^
16%	17.3	0.8 × 10^3^

**Table 5:** The Histopathological lesions in different treatments.

Lesions	Negative Control	Positive Control (Infected)	F2 (Oxytetracycline NPs Gel)	F3 (Commercial Eye Ointment)
Epithelial loss	**−**	+++	+	++
Inflammatory cells infiltration	**−**	+++	+	+
Edema and hyperemia	**−**	+++	+	++
Epithelial loss	**−**	+++	+	+
Inflammatory cells infiltration	**−**	+++	+	+
Edema	**−**	+++	++	++
Vascularization	**−**	+++	+	+
Depletion of pigmented cells	**−**	++	+	+
Leukocytic infiltration and edema	**−**	++	++	++

(**−**) = No lesion, (+) = Mild lesion, (++) = Moderate lesion, (+++) = Sever Lesion.

## Data Availability

The data presented in this study are available on request from the corresponding author.
